# AgrC biotinylation inhibits *Staphylococcus aureus* infection

**DOI:** 10.1371/journal.pone.0318695

**Published:** 2025-04-07

**Authors:** Lijuan Qian, Yuxin He, Wenzhe Lian, Zhiyuan Ji, Ziming Tian, Chuyun Wang, Chen Cao, Tyler Shern, Teagan Stedman, Yujun Sun

**Affiliations:** 1 College of Biomedicine and Health, Anhui Science and Technology University, Anhui, China; 2 College of Agriculture, Anhui Science and Technology University, Anhui, China; 3 BS-united China Group, International Genetically Engineered Machine (iGEM) Team, Anhui Science and Technology University, Anhui, China; 4 Columbia College, Columbia University, New York, United States of America; 5 Graduate School of Arts and Sciences, Columbia University Irving Medical Center, New York, United States of America; University of Houston, UNITED STATES OF AMERICA

## Abstract

*Staphylococcus aureus* (*S. aureus*) is a leading cause of nosocomial infections, particularly among antibiotic-resistant strains. *S. aureus* virulence is governed by the accessory gene regulator (Agr) quorum sensing (QS) system, which relies on AgrC, a two-component histidine kinase, to detect secreted auto-inducing peptides (AIPs). Emerging evidence highlights the potential of inhibiting the interaction between AgrC and AIPs as a promising therapeutic strategy. Given the limited clinic methods in inhibiting AgrC, we hereby report a novel method utilizing TurboID, an engineered biotin ligase, to inhibit Agr C on *S. aureus* via its biotinylation. To achieve this goal, a fusion protein named TurboID-AgrD_1−2_ (Agr-ID) was designed to include an AgrC binding domain (AgrID_1−2_) and a catalytic domain (TurboID) for AgrC biotinylation. By incubating with Alexa Fluor 647-conjugated streptavidin, the biotinylated AgrC on *S. aureus* was successfully visualized through fluorescence microscopy with 100x objective. We further confirmed the specific biotinylation of AgrC using Western Blotting, and biotinylated AgrC resulted in inhibiting the growth of *S. aureus* strains, including *S. aureus* 25923, *S. aureus* 43300, and *S. aureus* 6538 (MRSA). The downstream biological effect of AgrC biotinylation exhibited decreased virulence protein generation as monitored by the lower presence of apoptotic HEK 293T cells after incubating with *S. aureus* cell lysates and supernatant. The impaired colonizing features from biotinylated *S. aureus* 6538 were investigated by calculating the decreased ratio of cell death versus live HeLa cells. By further investigating the efficiency of the immune clearance of biotinylated *S. aureus* by mouse macrophages, we observed the enhanced uptake of *S. aureus* by murine macrophages *in vivo*. Overall, our work reveals that the biotinylation of AgrC can inhibit the growth and toxicity of *S. aureus* while simultaneously promoting the clearance of biotinylated *S. aureus* via macrophage phagocytosis.

## Introduction

*Staphylococcus aureus* (*S. aureus*) is a pathogenic bacterium that can cause a wide range of severe and potentially fatal infections, including bloodstream infections, pneumonia, skin and soft tissue infections and more. It has been identified as a leading cause of health-care-associated infections (HAIs) in the United States [1]. Over the past decade, *S. aureus* has been reported to have an annual incidence rate of 50 cases per 100,000 people with a mortality rate of 30% [2]. *S. aureus* has developed complex mechanisms to evade the human immune system, while also acquiring resistance to various commonly used classes of antibiotics. Among its most concerning forms is methicillin-resistant *S. aureus* (MRSA), which is associated with high morbidity and mortality rates, causing a significant public health burden. Currently, the primary clinically approved antibiotics for treating MRSA infections include vancomycin, daptomycin, linezolid, and ceftaroline [3]. However, the rise of multidrug-resistant MRSA, exacerbated by the overuse of antibiotics, has made treatment increasingly difficult. Quorum sensing (QS) is a cell-to-cell communication mechanism used by bacteria to regulate gene expression in response to population density, controlling various process such as virulence, biofilm formation, and antibiotic resistance [4]. In particular, QS is essential for the pathogenicity of *S. aureus*, and inhibiting QS can enhance its susceptibility to antibiotics by disrupting biofilm formation, a key mechanism contributing to antibiotic resistance [5]. Notably, the inhibition of AgrC, an autoinducer peptide (AIP)-sensing protein, has emerged as a promising therapeutic strategy for combating *S. aureus* infections. Previous studies have demonstrated that AgrC inhibitors, often structural analogs of AIPs, can prevent AIPs from binding to ArgC, thereby blocking the activation and phosphorylation of downstream AgrA. This disruption inhibits the subsequent production of RNAIII [6], a key regulator of virulence gene expression [7], and biofilm formation [8], ultimately weakening *S. aureus*’s resistance to antibiotics [9]. Although many of these inhibitors are rationally designed, overcoming the labile thiolactone bond in AIPs while maintaining their inhibitory activity remains a significant challenge [10,11]. Despite some challenges, AgrC inhibition continues to offer a promising therapeutic approach to modulate virulence and biofilm formation in *S. aureus*. Targeting the specific interaction between AgrC and AIPs, we introduce a novel approach by generating biotinylated AgrC using a novel tool called Agr-ID. Agr-ID is composed of an AgrC binding domain, named AgrD_1−2_, and a catalytic domain, TurboID. We further examined the successful labeling of biotinylated AgrC in *S. aureus*. Biotinylated AgrC enhanced cellular viability by approximately 20%, reduced *S. aureus* virulence by 10%, and increased the macrophage phagocytosis rate by 15% in the clearance of *S. aureus* *in vivo*.

## Materials and methods

### Materials

HEK293T cells and HeLa cells were purchased from the Chinese National Infrastructure of Cell Line Resource (NICR) and handled according to the instructions provided on the NICR product sheet. The *S. aureus* strains ATCC25923, *S. aureus* ATCC43300, and *S. aureus* ATCC6538 (MRSA) were purchased from the China Industrial Microbiological Culture Collection and Management Center. *Escherichia coli* (ATCC25922), *Pseudomonas aeruginosa* (ATCC27853), and *Bacillus sp* CZGRY11 were provided by the Horticulture Laboratory of Anhui Science and Technology University. Yeast powder was purchased from Oxoid Ltd. Peptone was purchased from the Beijing Aoboxing Biotechnology Co., Ltd. Imidazole and sodium chloride were purchased from the National Pharmaceutical Group Chemical Reagents Co., Ltd. Tris-HCl and agar powder were purchased from the Beijing Solebold Technology Co., Ltd. Polyacrylamide gels were purchased from the Nanjing Novozan Biotechnology Co., Ltd. Chloramphenicol and Isopropyl *β*-D-1-thiogalactopyranoside (IPTG) were purchased from Beyotime Biotech Inc. Nickel column fillers were purchased from Kingsley Biotechnology Co., Ltd.

### Ethics statement

Animal protocols were approved by the Institutional Animal Care and Use Committee at Anhui Science and Technology University (Animal protocol# AK2024039) and were performed in strict accordance with the Guide for the Use and Care of Laboratory Animals of the National Institutes of Health. At the end of the experiment, all mice were euthanized using carbon dioxide (CO_2_) gas. The mice were placed in an appropriate gas chamber and exposed to a gradually increasing concentration of CO_2_ (typically 30%–70%). The CO_2_ concentration was progressively raised to 70%, ensuring that the mice lost consciousness and entered a coma within 30 seconds. Following this, to ensure death, they were further exposed to CO_2_ for additional 2–3 min. To minimize distress, all euthanasia procedures were carried out in a quiet and warm environment. No anesthesia was administered prior to euthanasia, as exposure to CO_2_ is generally considered to cause minimal pain. Prior to euthanasia, the mice underwent an acclimatization period to the experimental environment, and efforts were made to ensure a calm atmosphere during the procedure. We implemented the following measures to reduce suffering in the mice during the experiment: Before the experiment began, the mice were allowed to acclimate to the experimental environment for at least 48 h to reduce stress from environmental changes. To minimize anxiety and pain, the rate of increase in CO_2_ concentration was controlled to not exceed 10%/min, ensuring that the mice lost consciousness quickly and avoiding prolonged exposure to the gas. After euthanasia, all animals underwent a post-mortem examination, such as cervical dislocation or cardiac puncture, to ensure no signs of consciousness recovery and to confirm death.

### Plasmid constructs

Agr-ID contains two domains. The catalytic domain gene sequence, TurboID-V5-6xHis, was obtained from AddGene (#107167) [12]. The AgrC binding domain, AgrD_1−2_, was designed and fused to the C-terminus of TurboID-V5-6xHis. The AIP region is located between amino acids 28 and 32 in AgrD_1−2_. In consideration of the protein folding and stability of Agr-ID, a PelB signal peptide was used to translocate the Agr-ID to the periplasm [13,14]. PelB-TurboID-V5-6xHis-AgrD_1−2_ (Agr-ID) gene was synthesized and cloned into pET-22b plasmid by the Beijing Genomics Institute (BGI). Flag tagged AgrD was constructed by inserting Flag tag after the first transmembrane domain (AgrID_6−28_) and cloned into pRN11(Addgene #84455) [15] by the Beijing Genomics Institute (BGI).

### Protein purification

The recombinant plasmid was transformed into BL21 (DE3) competent cells and plated on solid medium containing 100 µg/mL ampicillin. After 18 h, single colonies were selected for Sanger sequencing as part of the quality control (QC) process. The strains that passed the QC test were then cultured in 200 mL of Luria-Bertani (LB) medium at 37 °C and 200 rpm for approximately 4–6 h. After reaching an optical density at 600 nm (OD_600_) of 0.6, 200 µL of 1M Isopropyl *β*-D-1-thiogalactopyranoside (IPTG) was added to the medium, followed by continuous culturing at 16 °C for an additional 8 h. Finally, the supernatant was removed after centrifugation at 4 °C and 8000 rpm for 10 min. The pellet was then washed 3 times with 10 mL of 1 ×  phosphate buffered saline (PBS) before proceeding to lysis. Subsequently, 10 mL of a 5 mM imidazole solution was added to the collected bacteria, which were then subjected to ultrasonic crushing for 20 min using cycles of 9 s on and 6 s off. Bacterial lysates were then centrifuged at 4 °C and 10,000 rpm for 15 min. The QC for protein expression was assessed by conducting 10% SDS-PAGE followed by Coomassie staining. Ni+ column was pre-equilibrated by adding 10 mL of 5 mM imidazole and washing it for 3 times. Before passing through the Ni+ column, the supernatants were filtered using a 0.45 µm filter membrane. Ni+ based protein purification methods were followed by the protocol from Genscript. Agr-ID protein was kept in 50 mM Tris–HCl, pH 7.6, 150 mM NaCl at  − 80 °C.

### AgrC in situ biotinylation

0.45 mg/mL of Agr-ID was incubated with $∼\;1.66\;\times \;10_{9}$/mL *S. aureus* in 5 mM ATP, 25 mM MgCl_2_, and 50 mM Tris–HCl (pH 8.0) at 4 °C for 4 h for sufficient binding to AgrC. The biotinylation was initiated by adding 250 µM biotin at 30 °C for 30 min [12] and was terminated by adding 20-folded volume of 50 mM Tris–HCl at pH 8.0. The bacteria were then harvested by centrifuging at 3000 ×  g for 5 min.

### Fluorescence-based detection of biotinylated AgrC

*S. aureus* undergoing AgrC biotinylation was then incubated with 1 mM Alexa Fluor™ 647 Conjugate Streptavidin (Streptavidin-647) for 30 min. After washing with 50 mM Tris–HCl at pH 7.6, *S. aureus* was visualized using a Nikon fluorescent microscope equipped with a 100 ×  oil lens. *S. aureus* competent preparation and the electroporation were performed by following Dr. Richardson’s method [16]. Briefly, 1 µg pET9b-Flag-AgrC was mixed with *S. aureus* competent cells in 100 µL at room temperature for 5 min, then transferred into a 2 mm pulse cuvette (Bio-Rad). The waveform was set to exponential decay and pulse at 1.8 kV, 600 Ω, 10 *μ*F using the Gene Pulser Xcell Electroporation System (Bio-Rad). 500 µL LB medium was added immediately to resuspend the cells and then *S. aureus* were shaken at 37 °C for 1 h. All the cells were plated on ampicillin LB agar. The colonies were then ready for biotinylation and following detections. $∼\;1.66\;\times \;10_{9}$/mL biotinylated bacteria were pulled down using 30 µL anti-FLAG beads (Sigma M8823), followed by multiple washes with PBS and detection using a Multiskan SkyHigh plate reader (Thermo). The same treatment was performed on HEK293T cells, followed by Streptavidin-647 staining and detection of fluorescence intensity using the Multiskan SkyHigh plate reader (Thermo).

### Pulldown of biotinylated AgrC and Western Blotting

$∼\;1.66\;\times \;10_{9}$/mL biotinylated *S. aureus* were washed with PBS and centrifuged at 16,000 ×*g*. Pellets were lysed using pH 7.6 lysis buffer consisting of 20 mM Tris-HCl, 0.5 mM CaCl_2_, 50 mM NaCl, 40 *μ*g/mL DNase I, and 20 *μ*g/mL lysostaphin [17]. After centrifugation at 12,000 × g for 10 min, the supernatant was collected. 20-30 *μ*g/mL streptavidin-beads (Millipore, E5529) were added into the supernatant and incubate at 4 °C for 30 min. Beads were washed with PBS for 3 times first and 5 ×  SDS sample buffer was then added for a final concentration of 1 × . Samples were loaded onto a 15% SDS-PAGE gel, and proteins were transferred to a PVDF membrane before blocking with 5% milk. Streptavidin-HRP (Thermo Fisher, N100) was diluted into 1:5000 in Tris-Buffered Saline with 0.5% Tween 20 (TBST). The PVDF membrane was incubated with diluted Anti-FLAG antibody (HUABIO, M1403-2, 1:1000) for 1 h at room temperature, followed by 3 times wash with TBST and incubation with diluted secondary anti-mouse HRP antibody (HUABIO, HA1006, 1:5000). Imaging was performed by using a ChemiScope Mini chemiluminescence imaging system (Clinx Science).

### Bacterial growth

All the biotinylated *S. aureus* from the previous step were cultured in LB medium or on a LB agar plate. The density of *S. aureus* was assessed by measuring OD_600_ at 0, 2, 4, 8, and 18 h time points. The colonies were counted by plating  ∼ 500 *S. aureus* and culturing for 18 h. The same treatment was performed in *Escherichia coli*, *Pseudomonas aeruginosa*, and *Bacillus subtilis*.

### Detecting virulence protein level

The virulence proteins produced by *S. aureus* were measured by assessing the concentration of apoptotic co-cultured human HEK 293T cells [18]. $∼\;3\;\times \;10_{7}$ *S. aureus* cells were collected, and the *S. aureus* pellets were then lysed by ultrasound in 50 mM Tris–HCl at pH 7.6. 1 mL of supernatant and 1 mL of lysate were added separately to $1\;\times \;10_{6}$ HEK293T cells and incubated for 3 h. Apoptosis was detected by using the Annexin V kit (Thermo, A13201) and DAPI staining (Thermo, D1306), followed by detection via flow cytometry (BD Fortessa).

### Colonization

This protocol was modified from Xu et al, [19]. Briefly, *S. aureus* was pre-stained with 5 mg/mL Tetramethylrhodamine (TAMRA) Azide (Thermo, T10182) in PBS for 25 min. After washing 3 times with PBS, the *S. aureus* solution was diluted into  ∼ 10,000/mL, and 1000 *S. aureus* were added to 96 well plates with 10,000 HeLa cells in each well. After 1 h, the plates were washed 5 times with PBS to remove all detached *S. aureus*. HeLa cells were stained with Celltracker (Thermo, C7025) and checked by fluorescent microscopy at 60 ×  Len. Red dots colocalized with green cells were recognized as colonization.

### Mouse infection

This protocol was modified from Selina et al, [20]. Briefly, $1\;\times \;10_{6}$ TAMRA_+_ nonbiotinylated or biotinylated *S. aureus* were injected interperitoneally into 8-12 wks C57BL/6J mice and were then incubated for 15 min. After CO_2_ asphyxiation, peritoneal fluid was extracted, and all the cells were collected by centrifuging at 400 ×  g. Cells were blocked with 1 µg/mL CD16/32 antibody (Thermo, MA5-18012), followed by staining with 5 µg/mL anti-F4/80 antibody (Thermo, 53-4801-82). The macrophages with engulfed *S. aureus* were recognized by flow cytometry (BD Fortessa). For testing the combination therapy with vancomycin (Sigma, 1709007), we administered a 20 mg/kg intravenous injection of vancomycin into C57BL/6J mice 2 h before we performed the same mouse infection assay using $1\;\times \;10_{6}$ TAMRA_+_ biotinylated *S. aureus*.

### Statistical analysis

Statistical analysis was conducted using Graph Pad Prism 8. For *in vivo* studies, sample size calculation is performed in biomath (http://www.biomath.info/): using the phagocytes data in Fig. 3I to achieve a power of 0.9 at *α*=0.05, <6 mice per group are needed to detect a difference greater than 80% of the control mean with pooled standard deviation (SD) of 2.0 using two-tailed t-test. The data were first normalized through the Shapiro-Wilk test (N < 8). Data passing the normality test were then subjected to the two-tailed Students’ t-test for the comparison of two groups, one-way analysis of variance (ANOVA) or two-way ANOVA with Tukey’s multiple comparisons. The data passing the normality test were represented by the mean ± standard error of the mean (SEM). Two-sided P values were determined by the unpaired student t test for comparing data that did not pass the normality test. Data were then presented as mean ± SEM. Statistically significant differences were accepted with a P values less than 0.05. All the specific P values and biological replicates were listed in figures legends.

**Fig 1:**
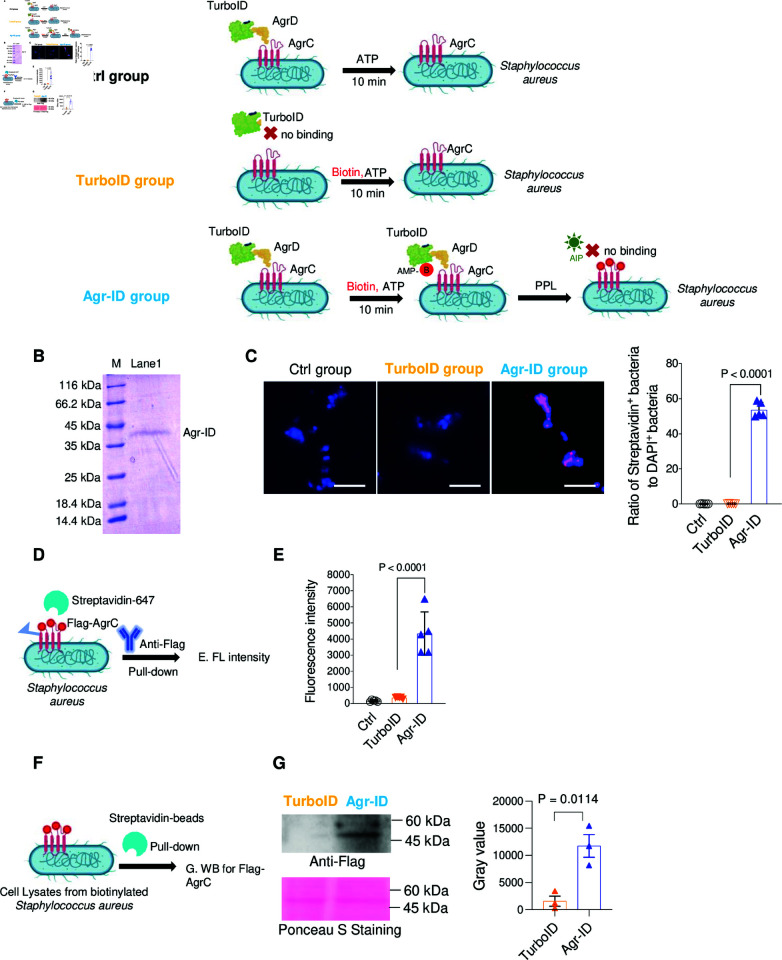
AgrC biotinylation by Agr-ID in *Staphylococcus aureus* (*S. aureus*). (A) Schematic model for the AgrC in situ biotinylation. The control group lacks biotin treatment. TurboID group represents none specific biotinylation without AgrC binding domain. Agr-ID is the experimental group targeting the biotinylation of AgrC. (B) Purity of Agr-ID analysis by SDS -10% PAGE and Coomassie blue staining. (C) Visualization of the biotinylated *S. aureus* by co-staining with Streptavdin-647. The percentage of biotinylated *S. aureus* was qualified by calculating the ratio of Streptavidin_+_ *S. aureus* 25923 versus total DAPI_+_ *S. aureus* 25923. N=5 independent biological replicates. (D-E) Pull-down assay for detecting the AgrC in situ biotinylation. Flag-agrC was transformed and overexpressed into *S. aureus*. The average fluorescent intensity of biotinylated AgrC was measured in all *S. aureus* pulled down by anti-Flag beads. N=5 independent biological replicates. (F-G) Pull-down assay for detecting the AgrC protein biotinylation using Western Blotting. Biotinylated proteins were pulled down by streptavidin-beads and analyzed by Western Blotting for detecting FLAG-AgrC. N=3 independent biological replicates. Data are presented as mean ± SEM. Two-sided P values were determined by unpaired student t-test. Data are presented as mean ± SEM. Two-sided P values were determined by a one-way ANOVA with Tukey’s multiple comparisons test.

**Fig 2:**
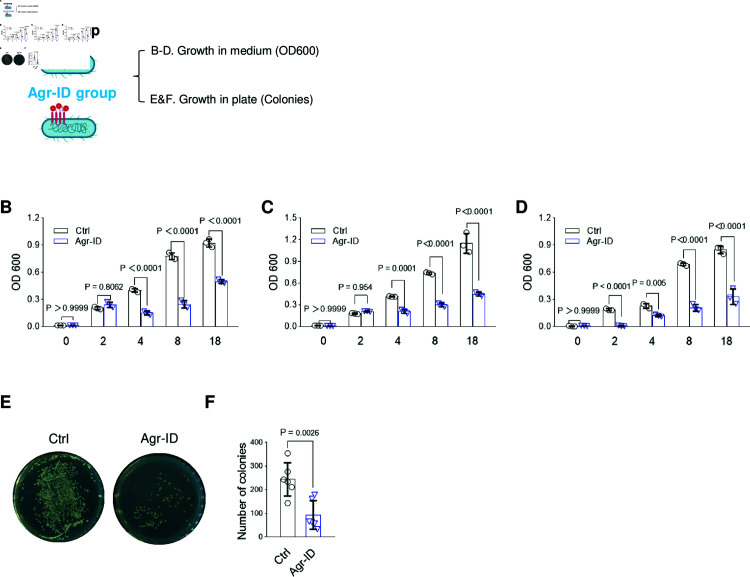
AgrC biotinylation by Agr-ID inhibited the growth of *S. aureus*. (A) Schematic model for testing the growth of biotinylated *S. aureus* in LB medium and LB agar plate. (B-D) Growth inhibition in biotinylated *S. aureus* 25923 (B), *S. aureus* 6538 (C), *S. aureus* 43300 (D). $∼\;1.66\;\times \;10_{6}$/mL biotinylated *S. aureus* were added to 5 mL LB medium and cultured at 37 °C. At each time points, 1 mL medium was taken out for detecting the OD_600_, then mixed back with the original ones. N=3 independent biological replicates. (E&F) Less colonies were shown in biotinylated S. aureus 25923. N=6 independent biological replicates. Data are presented as mean ± SEM. Two-sided P values were determined by unpaired student t-test. Data are presented as mean ± SEM. Two-sided P values were determined by a two-way ANOVA with Tukey’s multiple comparisons test.

## Results

### The construction and characterization of Agr-ID

Dr. Ting’s lab developed the engineered biotin ligase, named TurboID [21], and they have previously performed efficient proximity labeling in living cells and organisms [12]. By leveraging this tool for biotinylating *S. aureus*, we generated a fusion protein named Agr-ID for creating biotinylated AgrC. As shown in Fig 1A, Agr-ID was equipped with an AgrC binding domain, AgrD_1−2_, and a catalytic domain, TurboID. AgrD_1−2_, which contains the autoinducer peptide (AgrD_28−32_) at its C-terminus, first anchors to AgrC, bringing TurboID closer in proximity to AgrC. Under the short exposure to biotin and ATP over 30 min, TurboID can facilitate the biotinylation of exposed lysine residues within a 10 nm radius by the effective conversion of ATP and biotin to form a reactive biotinoy1-5’-AMP conjugate [21]. We purified the Agr-ID protein to perform the biotinylation of *S. aureus* and we checked the purity by running SDS-10% PAGE, followed by Coomassie Blue Staining (Fig 1B). The biotinylated AgrC can be visualized by the incubation with Alexa Fluor™ 647 conjugated streptavidin (streptavidin-647), followed by detection using fluorescence microscope (Fig 1C). Streptavidin_+_ *S. aureus* was measured to be around 60%, indicating the successful binding and catalytic activity in converting ATP and biotin to form a reactive biotinoy1-5’-AMP conjugate. To further confirm the biotinylation of AgrC, we delivered a Flag-tagged agrC gene into *S. aureus* (Fig 1D) and performed the same assay shown in Fig 1A. After pulling down all the FLAG tagged *S. aureus* using the anti-FLAG antibody, FLAG-AgrC was successfully labeled with biotin as shown by the significant increase of fluorescent intensity (Fig 1E). In order to confirm the biotinylation of AgrC, we lysed the biotinylated *S. aureus* expressed with Flag-agrC and detected the FLAG-AgrC protein using Western blotting (Fig 1F). The band shown in Fig 1G corresponds to FLAG-AgrC ( ∼ 50 kDa), indicating that the entire biotin-labeled protein pool generated by pull-down with streptavidin beads includes FLAG-AgrC.

### Agr-ID inhibited the growth of *S. aureus* 25923, *S. aureus* 43300, and *S. aureus* 6538 (MRSA)

To further investigate biotinylated AgrC in inhibiting the growth of *S. aureus*, we then measured the bacterial growth curve by detecting OD_600_ (Fig 2A), the most commonly used technique to monitor the growth of bacteria [22]. As shown in Fig 2B, there was no significant difference in the first 2 h, indicating that the biotinylated *S. aureus* 25932 exhibited minimal death and was not dying in the first 2 h. However, the growth inhibition showed a significant difference between the two groups (Ctrl vs Agr-ID), suggesting that the blocking of surface AgrC protein by Agr-ID resulted in growth arrest after 2 h. Additionally, it is known that biofilm-associated genes are usually activated when the OD_600_ reaches 0.8 [23]. We observed that the growth of biotinylated *S. aureus* 25932 did not reach an OD_600_ value of 0.8, indicating reduced QS signaling and inhibited biofilm production in this strain. To further confirmed the growth inhibition of biotinylating on other strains of *S. aureus*, we employed two additional strains: including *S. aureus* 6538 (Fig 2C) and *S. aureus* 43300 (Fig 2D). We also confirmed growth inhibition by counting the colonies on the LB agar plate, which showed a 60% decrease of colony numbers in the biotinylated group (Figs 2E and 2F). Under the biotinylating conditions, both *S. aureus* strains exhibited growth arrest, indicating the successful biotinylation of the surface AgrC proteins on *S. aureus* 43300 and suggesting the potential use of this method for anti-bacterial treatments.

**Fig 3:**
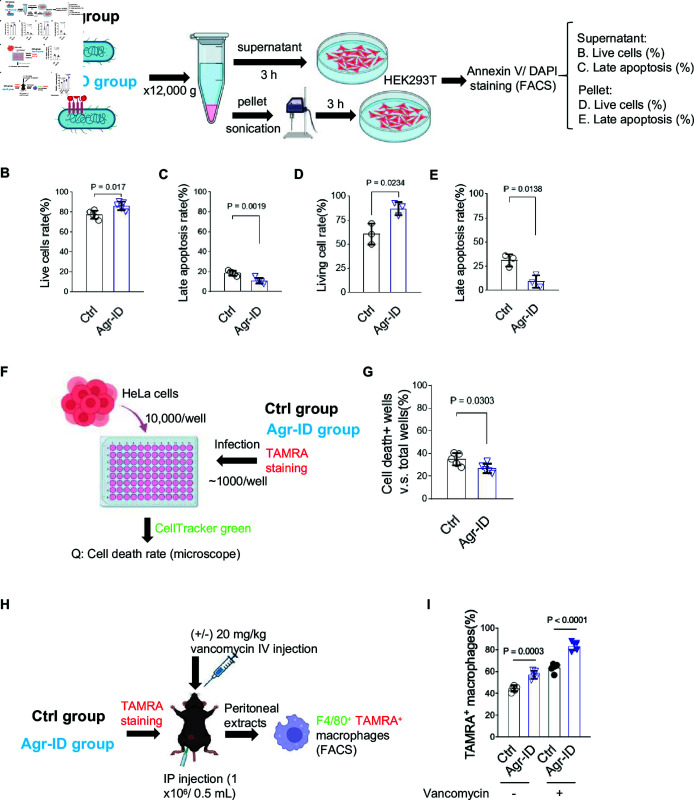
AgrC biotinylation by Agr-ID inhibited the virulence proteins of *S. aureus* and promoted phagocytes clearance of *S. aureus* *in vivo*. (A) Schematic model for testing the virulence factors of biotinylated *S. aureus*. (B-E) Virulence proteins from biotinylated *S. aureus* 25923 led to less cell death. Less virulence protein in supernatant (B&C) and cell lysates (D&E) after incubating with HEK293T cells. The percentage of apoptotic HEK293T cells was detected and measured by Annexin V/DAPI staining and FACS. N=3 independent biological replicates. (F&G) Impaired colonization of biotinylated *S. aureus* 25923. The percentage of cell death_+_ was counted by co-localization of TAMRA and CellTracker under fluorescent microscope. N=5 independent biological replicates. (H&I) Biotinylated *S. aureus* 25923 had impaired capacity in infecting mice. The enhanced percentage of TAMRA_+_ macrophages showed more efficiency in clearance of biotinylated *S. aureus* 25923 by macrophages. N=5 independent biological replicates. Data are presented as mean ± SEM. Two-sided P values were determined by unpaired student t-test. Data are presented as mean ± SEM. Two-sided P values were determined by a two-way ANOVA with Tukey’s multiple comparisons test.

### Agr-ID reduced the virulence of *S. aureus* 25923 and impaired colonization

To further investigate the production of virulence proteins in *S. aureus* 25923, both lysates and supernatant were utilized to assess their ability to induce apoptosis in mammalian cells [18]. After 18 h of culture, equal numbers of non-biotinylated and biotinylated *S. aureus* 25923 were harvested (Fig 3A). Both virulence proteins levels in *S. aureus* and the supernatant were lower as reflected by increased live cells and decreased apoptotic cells in the biotinylated group (Figs 3B–3E). The results indicated a decreased QS signaling in biotinylated *S. aureus* 25923. The accessory gene regulator is a highly conserved yet polymorphic QS that plays a crucial role in colonization [24]. To investigate whether the biotinylated AgrC will further impair colonization in *S. aureus*, we then performed the infection of HeLa cells by non-biotinylated and biotinylated *S. aureus* 25923. Impaired colonization in biotinylated *S. aureus* was shown by decreased cell death (Figs 3F and 3G). Moreover, Agr-mediated QS signaling impaired host defense against *S. aureus* [25]. To further test the recognition by host primary macrophages, TAMRA labeled non-biotinylated and biotinylated *S. aureus* 25923 were injected interperitoneally into mice (Fig 3H). Enhanced phagocytosis by macrophages were seen in biotinylated *S. aureus* 25923 group, indicating that the inhibition of AgrC can facilitate the host primary macrophages to perform the clearance of the biotinylated *S. aureus* (Fig 3I). These results reflected that biotinylated *S. aureus* 25923 was less toxic compared to non-biotinylated *S. aureus* 25923.

## Discussion

This study reveals that biotinylated AgrC exerts substantial detoxification of *S. aureus* strains via Agr-ID. The non-toxic treatment with Agr-ID, along with biotin and ATP, shows great therapeutic potential in inhibiting *S. aureus* infections. TurboID has widely been used as an effective tool for investigating novel and transient interactomes [21,26,27] due to its high biotin ligase activity. Tess et al, reported the first study to initiate promiscuous biotinylation in yeast [12]. However, it remains unknown whether biotinylation of yeast surface proteins affects cell proliferation, cell-cell communication, and metabolism.

Biotinylating cell surface proteins is widely used in cell engineering to control cellular interactions [16]. By covalently attaching biotin to membrane proteins via amine groups, followed by streptavidin-biotin binding to nanomaterials, this system shows strong potential for multifunctional drug delivery. It has also been applied to functionalize mesenchymal stem cells (MSCs) with Sialyl Lewis X (SLeX) [28], where covalent SLeX conjugation via a biotin-streptavidin bridge promotes cell rolling [28]. However, a more specific biotinylation technique is recommended to replace the less specific chemical biotinylation method [29]. In our current study, the focus is on specifically labeling AgrC with biotin in *S. aureus* (Fig 1). This approach could facilitate the conjugation of streptavidin with drugs to selectively target and kill *S. aureus*.

A simple biotin labeling on protein may result in minimal changes to its enzymatic activity, but biotinylation of lysine changes its charge. The loss of positive charge due to biotinylation of lysine can significantly affect its interaction with substrates or binding partners, especially when the substrates are small peptides. The biotinylation of AgrC may change the charge-dependent interactions, hydrogen bonding, and introduce steric hindrance.

Our work aimed to uncover the effects of the biotinylation of AgrC on *S. aureus*. Agr-ID was designed to target the surface protein AgrC on *S. aureus*, and we further confirmed the biotinylation on Flag-AgrC using a pull-down strategy (Fig 1D–1G). Since we noticed that the biotinylation of AgrC impaired the growth of *S. aureus* (Fig 2), which is an indirect consequence in addition to QS, it has been reported that the agr locus is essential for colony spreading [30]. Biotinylation of AgrC resulted in inhibition of virulence gene expression (Fig 3A–3G), indicating that the silencing of the agr locus by biotinylation of AgrC may affect the decreased glucose-promoted colony spreading and growth. However, biotinylation is not limited to AgrC alone; it also biotinylates its nearby proteins [12]. This could result in blocking other signal peptides from crossing the *S. aureus* cell wall, such as the YSIRK/GXXS signal peptide [31]. Further studies can be done to reveal more detailed modifications of its client proteins by pulling down all biotinylated proteins and performing mass spectrometry analysis.

Our research highlights that Agr-ID can only inhibit and detoxify *S. aureus* through biotinylating AgrC. The nonspecific binding of Agr-ID was further confirmed by adding Agr-ID and ATP, with or without biotin treatment, to HEK293T cells (S1A Fig). Agr-ID had less unexpected binding to HEK293T cell, as indicated by a lack of significant difference in fluorescent intensity between biotin treated and none-treated groups (S1B Fig). Moreover, Agr-ID also showed high specificity when tested on other bacteria, such as *Escherichia coli* (S2A Fig), *Pseudomonas aeruginosa* (S2B Fig), and *Bacillus subtilis* (S2C Fig). No significant changes in colony numbers on LB plates indicated the success of Agr-ID in specifically biotinylating *S. aureus*. To further explore the interaction interface between Agr-ID and AgrC, we modeled the Agr-ID: AgrC_206−430_ complex using the AlphaFold 3 Webserver [32]. As labeled in S3 Fig, the C-terminal lobes of Agr-ID were predicted to format one electrostatic bond and one hydrophobic bond with AgrC_206−430_, further supporting the potential specificity of Agr-ID.

There are still unanswered questions about the appropriate dosage of Agr-ID needed to rescue an animal in preclinical studies. In this study, we claimed that 15% more peritoneal macrophages are capable of clearing the injected biotinylated *S. aureus* (Fig 3H and 3I). It remains unknown whether Agr-ID proteins can efficiently label *S. aureus* and rescue an *S. aureus*-infected mouse.

## Supporting information

S1 Fig HEK293 cells were not biotinylated by Agr-ID. (A) Agr-ID and ATP, with or without biotin, were incubated with HEK293T cells for 30 min, followed by co-staining with Streptavidin-647 as indicated. (B) The quantification was measured by detecting the averaged fluorescent intensity using plate reader. N=5 independent biological replicates as indicated. Data are presented as mean ± SEM. Two-sided P values were determined by unpaired student t test. Data are presented as mean ± SEM. Two-sided P values were determined by a two-way ANOVA with Tukey’s multiple comparisons test.
(TIF)

S2 Fig Other bacterial species were not biotinylated by Agr-ID. Agr-ID and ATP, with or without biotin, were incubated with *Escherichia coli* (A), *Pseudomonas aeruginosa* (B), and *Bacillus subtilis* (C) for 30 min. The capacity was measured by counting the colonies on LB plates. N=5 independent biological replicates as indicated. Data are presented as mean ± SEM. Two-sided P values were determined by unpaired student t test. Data are presented as mean ± SEM. Two-sided P values were determined by a two-way ANOVA with Tukey’s multiple comparisons test. (TIF)

S3 Fig Structural insights into the interaction between Agr-ID and hydrophobic domain AgrC_206−430_. Agr-ID and AgrC_206−430_ complex were modeled with AplhaFold 3 Webserver. The predicted complex structure indicated the C-terminal lobes of Agr-ID contributed to the interaction with AgrC_206−430_. Electrostatic bond was formatted between Lys67 and Asp361 while hydrophobic bond was formatted between Phe359 and Leu40. (TIF)

S1 File Raw data. (DOCX)

## Acknowledgments

We would like to express our sincere gratitude for the financial support provided by the Natural Science Research Major Funding Project of Anhui Universities (KJ2021ZD0107), which has enabled us to conduct experiments and analyze data.
