# Supplementary figures and images for: AgrC biotinylation inhibits Staphylococcus aureus infection
Source: PLoS One. 2025 Apr 7;20(4):e0318695. doi: 10.1371/journal.pone.0318695 (PMC11991674; doi:10.1371/journal.pone.0318695)

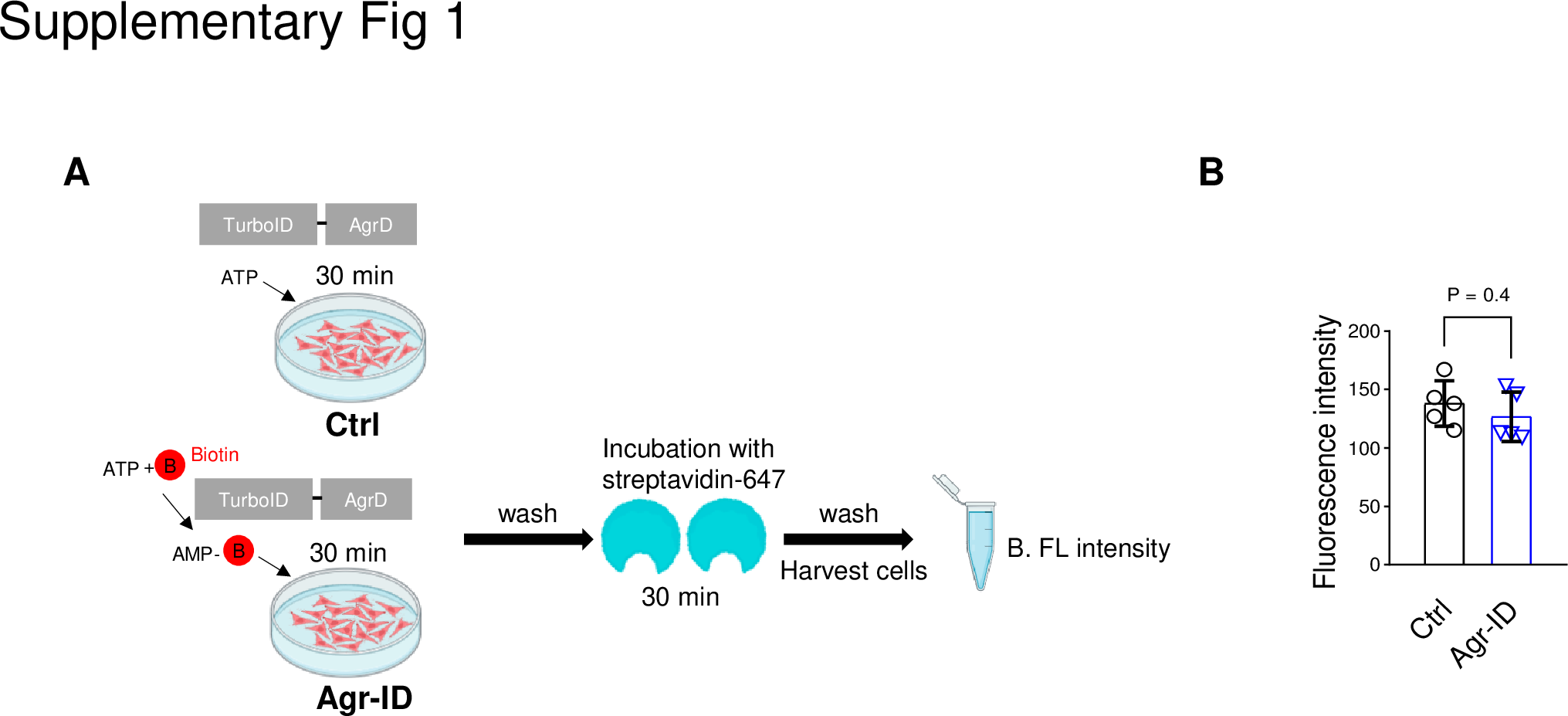

Supplement: S1 Fig — (TIF) [file pone.0318695.s001.tif]

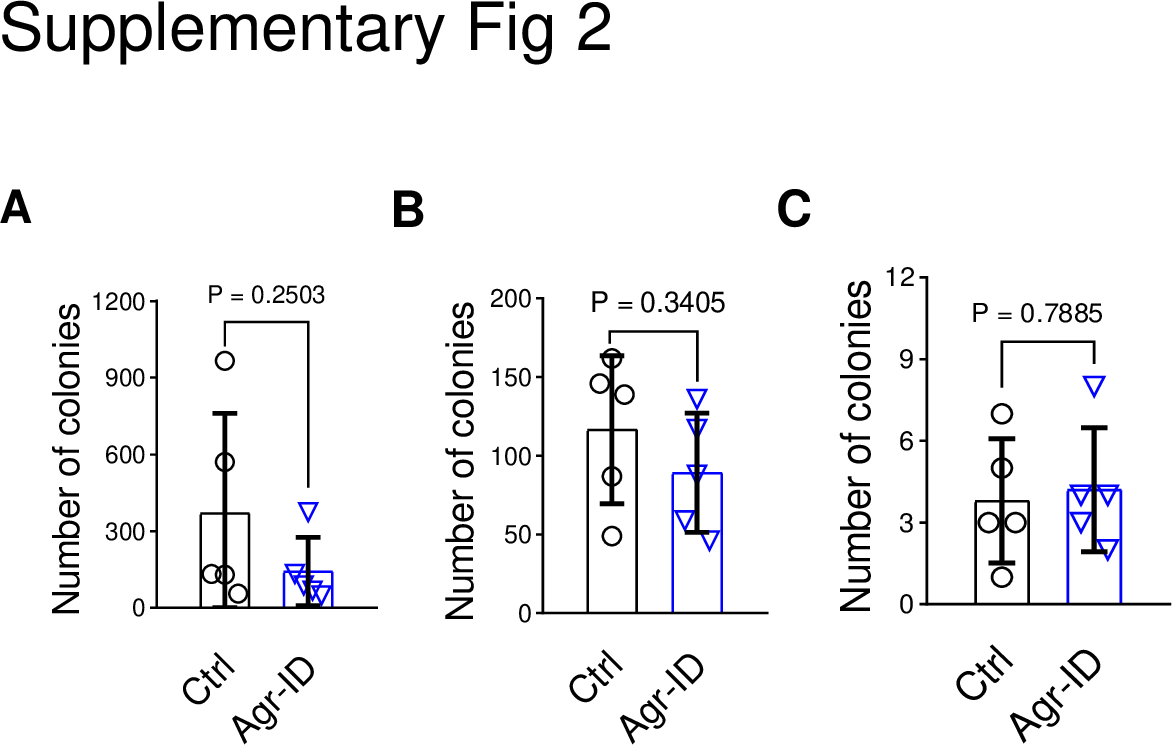

Supplement: S2 Fig — (TIF) [file pone.0318695.s002.tif]

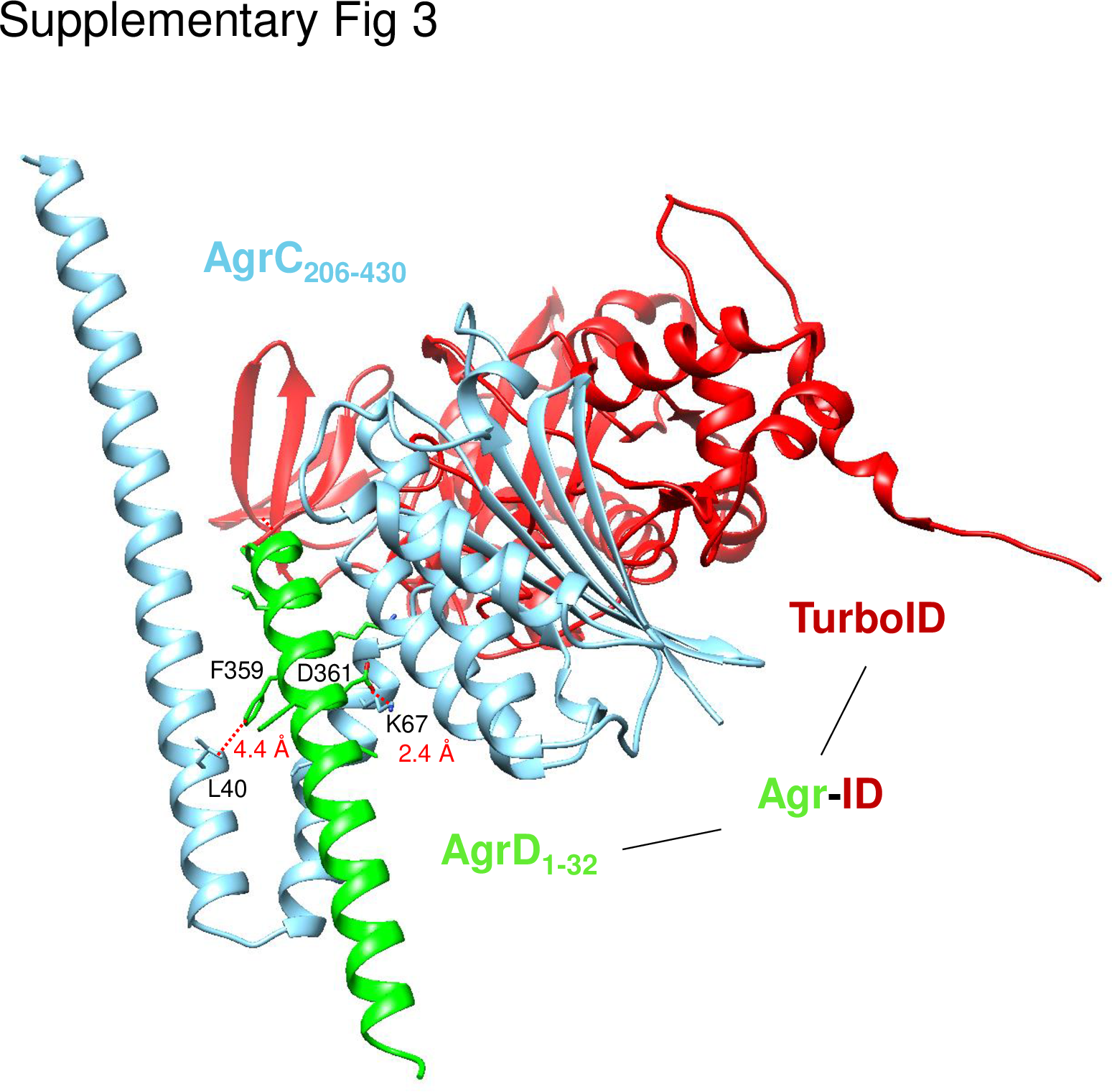

Supplement: S3 Fig — (TIF) [file pone.0318695.s003.tif]
