# Supplementary material for: AgrC biotinylation inhibits Staphylococcus aureus infection
Source: PLoS One. 2025 Apr 7;20(4):e0318695. doi: 10.1371/journal.pone.0318695 (PMC11991674; doi:10.1371/journal.pone.0318695)
Supplement: S1 File — (DOCX) [file pone.0318695.s004.docx]

AgrID sequence:

ATGAAATACCTGCTGCCGACCGCTGCTGCTGGTCTGCTGCTCCTCGCTGCCCAGCCGGCGATGGCCATGGGGgccagcaaagacaatactgtgcctctgaagctgatcgctctcctggctaatggcgagttccatagtggcgaacagctgggagaaaccctgggcatgtccagggccgctatcaacaagcacattcagactctgcgcgactggggcgtggacgtgttcaccgtgcccggaaagggctactctctgcccgagcctatcccgctgctgaacgctaaacagattctgggacagctggacggcgggagcgtggcagtcctgcctgtggttgactccaccaatcagtacctgctggatcgaatcggcgagctgaagagtggggatgcttgcattgcagaatatcagcaggcagggagaggaagcagagggaggaaatggttctctccttttggagctaacctgtacctgagtatgttttggcgcctgaagcggggaccagcagcaatcggcctgggcccggtcatcggaattgtcatggcagaagcgctgcgaaagctgggagcagacaaggtgcgagtcaaatggcccaatgacctgtatctgcaggatagaaagctggcaggcatcctggtggagctggccggaataacaggcgatgctgcacagatcgtcattggcgccgggattaacgtggctatgaggcgcgtggaggaaagcgtggtcaatcagggctggatcacactgcaggaagcagggattaacctggacaggaatactctggccgctacgctgatccgagagctgcgggcagccctggaactgttcgagcaggaaggcctggctccatatctgccacggtgggagaagctggataacttcatcaatagacccgtgaagctgatcattggggacaaagagattttcgggattagccgggggattgataaacagggagccctgctgctggaacaggacggagttatcaaaccctggatgggcggagaaatcagtctgcggtctgccgaaaagCACCACCACCACCACCACATGAATACATTATATAAATCATTTTTTGATTTTATAACTGGTGTTTTAAAAAACATTGGTAACGTTGCTTCTTATAGTACATGTTATTTCATAATGTAA

Flag-AgrC sequence:

atggtccaaactagtatggaattgttaaacagttacaactttgttttgttcgtattaactcaaataatactaatgtttacagtaccatcaattattagtggtGATTACAAAGATGACGATGACAAAatcaaatacagtaaatctgactatttgtatatcacgggtattacggcattatcgttaattctatttaattttatcgatagcgtaaccttaataatactaactatattcataattatattatatctcagcaaaatcaaatggtattctattttgctgataatgacctcgcagattattttatactgtgcaaattacatgtatatagttatattcacatatattgtcaaaatcgttgatagtatatttgtaatatttcccatcttctttgtagtttatgtgacaatcagtatactattttcttatataataaatagagttctcaaaaagattagctcaacatatctaatactaaacaaaggttttttaatagttatttcaaccattctactgcttactttttcattgtttttcttttattctcaaataaactcggatgaagctaaagtaataaggcagtattcttttatttttattggaatcactatatttttaagtatattaacatttgttatttcacaatttctctttaaagagatgaaatacaaaagaaatcaagaagaaattgagacctattacgaatacacattaaagattgaagcaattaataatgaaatgcgtaagttccgacatgattatgtcaatatcttaactacactttcagaatacattcgagaagatgacatgcctggtctacgtgactatttcaataaaaatatagtgccaatgaaagataacttacaaatgaacgctattaagttaaatggtattgagaatcttaaagtacgtgaaattaaaggcttaatcactgctaaaattttacgtgcacaagaaatgagtataccgattagtattgaaataccagatgaagtaactcacattaatttgaatatgattgatttaagtcgcagtattggtattattcttgataacgcaatagaggcatcaactgaaattgatgatcctatcattcgagttgcatttattgaaagcgaaaattcagtaacgtttattgttatgaataaatgtgcagatgatataccacgtattcatgaattgttccaagaaagtttttctactaaaggtgaaggtcgtggtttaggtctatcaactttaaaagaaattgctgataatgcagacaatgtcttattagatacaattatcgagaatggtttctttattcaaaaggttgaaattattaacaactaa

Figure 1B


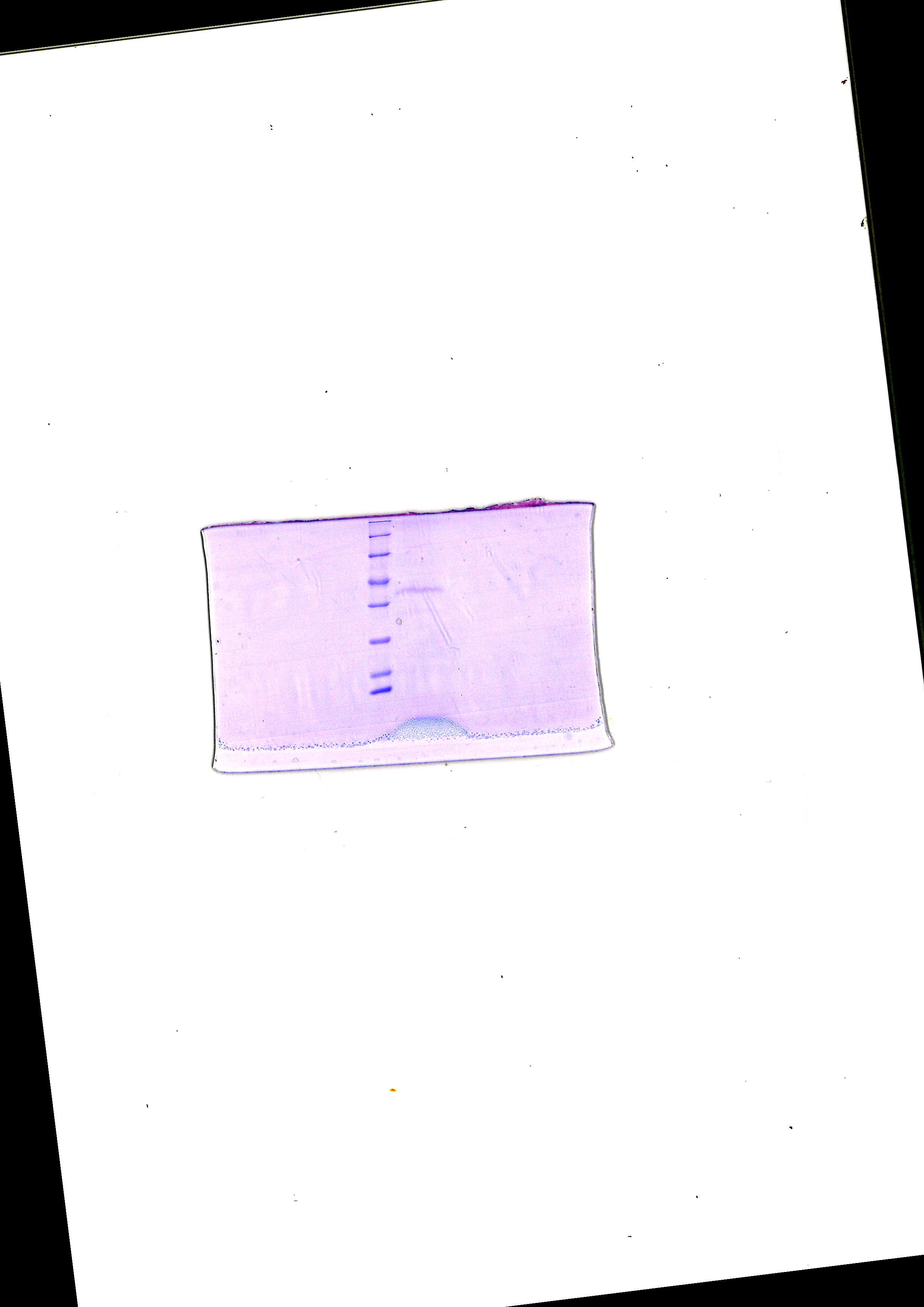


Agr-ID

14.4 kDa

18.4 kDa

25 kDa

35 kDa

45 kDa

116 kDa

66.2 kDa

M

Figure 1C

| # | Ratio of Streptavidin+ bacteria to DAPI+ bacteria | | |
| --- | --- | --- | --- |
|  | Ctrl | TurboID | Agr-ID |
| 1 | 0.1 | 0.1 | 50.0 |
| 2 | 0.2 | 0.1 | 58.9 |
| 3 | 0.1 | 0.2 | 57.9 |
| 4 | 0.1 | 0.1 | 51.2 |
| 5 | 0.1 | 0.3 | 50.7 |

Figure 1E

| # | Fluorescence intensity | | |
| --- | --- | --- | --- |
|  | Ctrl | TurboID | Agr-ID |
| 1 | 115 | 360 | 3200 |
| 2 | 112 | 375 | 4500 |
| 3 | 245 | 266 | 4300 |
| 4 | 180 | 401 | 3200 |
| 5 | 177 | 387 | 6500 |

Figure 1G

TurboID


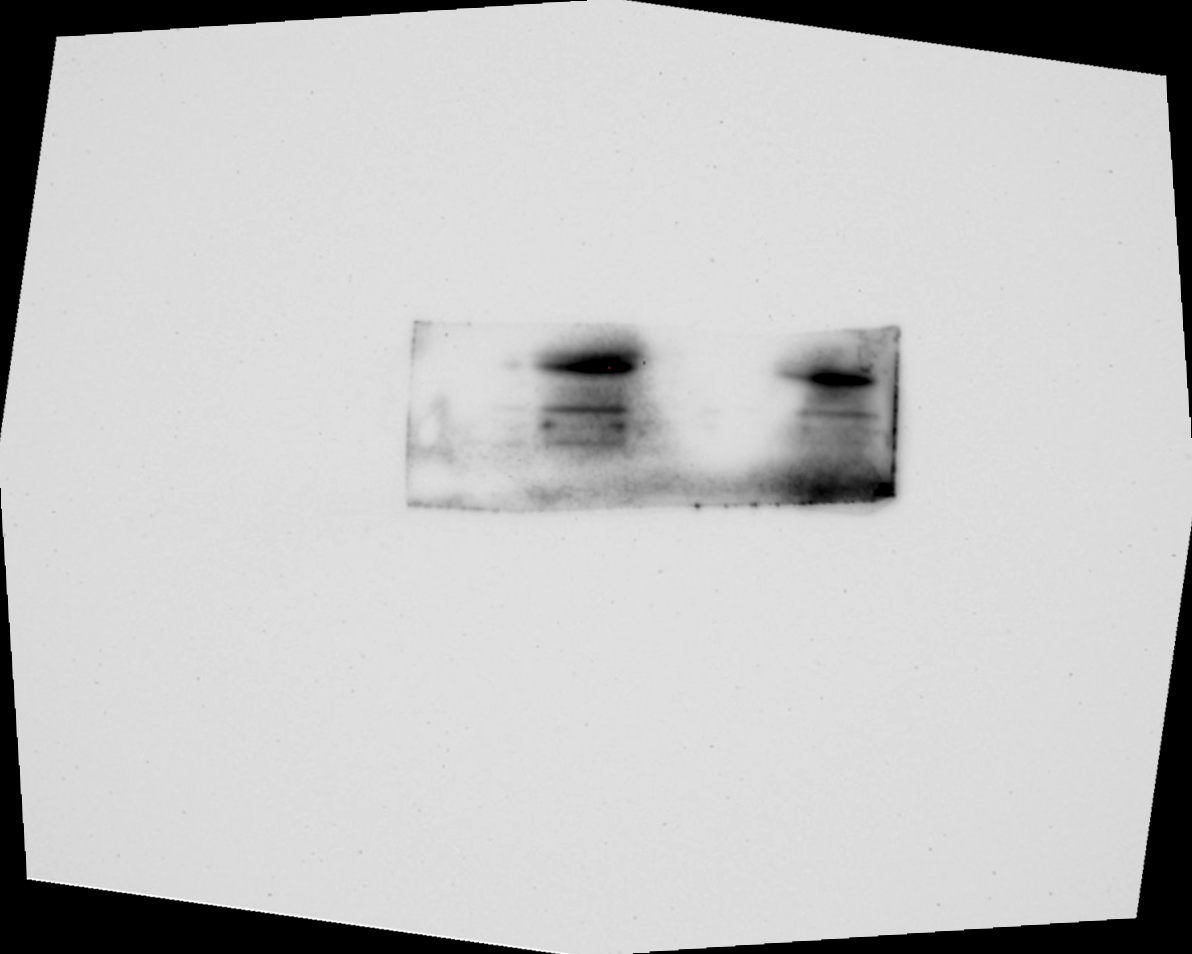


Agr-ID

Chemiluminescence (Western blotting)


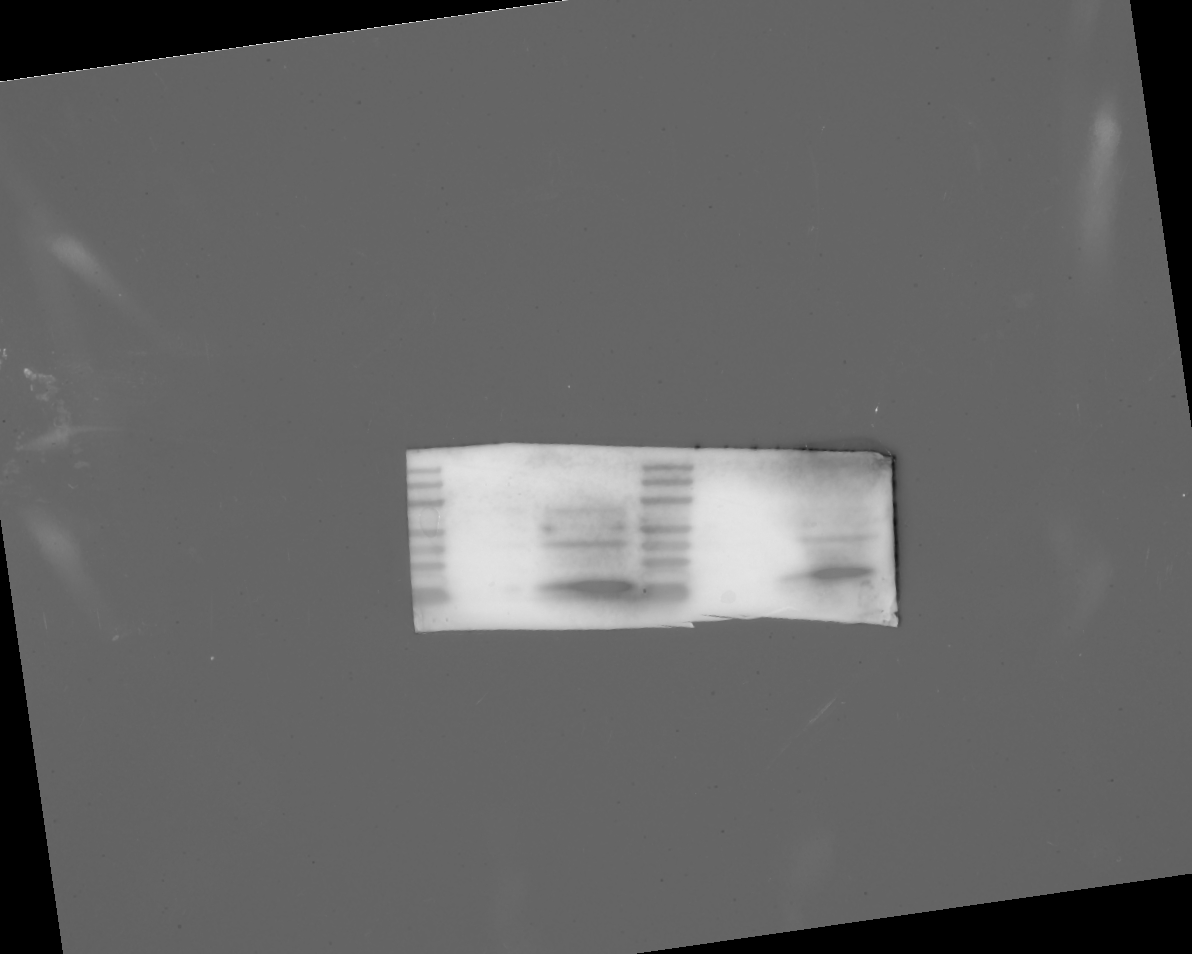


Agr-ID

10 kDa

15 kDa

25 kDa

35 kDa

45 kDa

60 kDa

75 kDa

M

TurboID

100 kDa

140 kDa

180 kDa

Bright Field merged with Chemiluminescence (Western blotting)


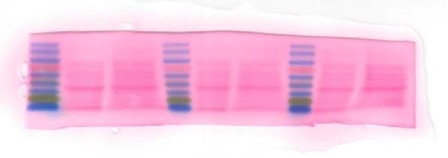


Agr-ID

TurboID

60 kDa

180 kDa

140 kDa

100 kDa

10 kDa

15 kDa

25 kDa

35 kDa

45 kDa

75 kDa

M

Ponceau S staining

| # | Gray value | |
| --- | --- | --- |
|  | TurboID | Agr-ID |
| 1 | 1098.68 | 15398.93 |
| 2 | 3361.96 | 11689.91 |
| 3 | 228.66 | 8138.96 |

Figure 2B

| Time / h | OD 600 | | | | | | | |
| --- | --- | --- | --- | --- | --- | --- | --- | --- |
|  | Ctrl-1 | Ctrl-2 | Ctrl-3 | Ctrl-avg | Agr-ID-1 | Agr-ID-2 | Agr-ID-3 | Agr-ID-avg |
| 0 | 0.014 | 0.012 | 0.014 | 0.013 | 0.013 | 0.012 | 0.014 | 0.013 |
| 2 | 0.220 | 0.190 | 0.200 | 0.203 | 0.240 | 0.270 | 0.210 | 0.240 |
| 4 | 0.404 | 0.378 | 0.414 | 0.399 | 0.152 | 0.127 | 0.168 | 0.149 |
| 8 | 0.817 | 0.745 | 0.768 | 0.777 | 0.243 | 0.207 | 0.289 | 0.246 |
| 18 | 0.926 | 0.877 | 0.965 | 0.923 | 0.520 | 0.478 | 0.491 | 0.496 |

Figure 2C

| Time / h | OD 600 | | | | | | | |
| --- | --- | --- | --- | --- | --- | --- | --- | --- |
|  | Ctrl-1 | Ctrl-2 | Ctrl-3 | Ctrl-avg | Agr-ID-1 | Agr-ID-2 | Agr-ID-3 | Agr-ID-avg |
| 0 | 0.009 | 0.011 | 0.013 | 0.011 | 0.003 | 0.004 | 0.007 | 0.005 |
| 2 | 0.180 | 0.183 | 0.170 | 0.178 | 0.210 | 0.200 | 0.210 | 0.207 |
| 4 | 0.420 | 0.410 | 0.410 | 0.413 | 0.230 | 0.210 | 0.180 | 0.207 |
| 8 | 0.720 | 0.750 | 0.740 | 0.737 | 0.320 | 0.270 | 0.300 | 0.297 |
| 18 | 1.030 | 1.120 | 1.300 | 1.150 | 0.420 | 0.450 | 0.470 | 0.447 |

Figure 2D

| Time / h | OD 600 | | | | | | | |
| --- | --- | --- | --- | --- | --- | --- | --- | --- |
|  | Ctrl-1 | Ctrl-2 | Ctrl-3 | Ctrl-avg | Agr-ID-1 | Agr-ID-2 | Agr-ID-3 | Agr-ID-avg |
| 0 | 0.003 | 0.001 | 0.009 | 0.004 | 0.004 | 0.008 | 0.009 | 0.007 |
| 2 | 0.180 | 0.180 | 0.200 | 0.187 | 0.013 | 0.002 | 0.004 | 0.006 |
| 4 | 0.200 | 0.230 | 0.250 | 0.227 | 0.120 | 0.130 | 0.110 | 0.120 |
| 8 | 0.670 | 0.700 | 0.690 | 0.687 | 0.180 | 0.250 | 0.190 | 0.207 |
| 18 | 0.870 | 0.870 | 0.800 | 0.847 | 0.320 | 0.250 | 0.420 | 0.330 |

Figure 2F

| # | Number of colonies | |
| --- | --- | --- |
|  | Ctrl | Agr-ID |
| 1 | 234 | 57 |
| 2 | 354 | 63 |
| 3 | 276 | 65 |
| 4 | 211 | 161 |
| 5 | 143 | 33 |
| 6 | 243 | 178 |

Figure 3B

| # | Live cells rate (%) | |
| --- | --- | --- |
|  | Ctrl | Agr-ID |
| 1 | 71.5 | 87.1 |
| 2 | 75.2 | 83.4 |
| 3 | 80.1 | 80.3 |
| 4 | 82.3 | 89.4 |
| 5 | 77.3 | 90.5 |

Figure 3C

| # | Late apoptosis rate (%) | |
| --- | --- | --- |
|  | Ctrl | Agr-ID |
| 1 | 21.4 | 10.5 |
| 2 | 19.8 | 11.2 |
| 3 | 17.3 | 15.3 |
| 4 | 15.0 | 9.8 |
| 5 | 19.0 | 7.5 |

Figure 3D

| # | Living cell rate (%) | |
| --- | --- | --- |
|  | Ctrl | Agr-ID |
| 1 | 71.6 | 89.0 |
| 2 | 60.5 | 79.4 |
| 3 | 50.0 | 92.6 |

Figure 3E

| # | Late apoptosis rate (%) | |
| --- | --- | --- |
|  | Ctrl | Agr-ID |
| 1 | 23.80 | 8.03 |
| 2 | 33.43 | 16.09 |
| 3 | 35.94 | 3.53 |

Figure 3G

| # | Cell death+ wells v.s. total wells(%) | |
| --- | --- | --- |
|  | Ctrl | Agr-ID |
| 1 | 31.3 | 33.1 |
| 2 | 35.2 | 28.7 |
| 3 | 27.8 | 25.6 |
| 4 | 39.6 | 24.5 |
| 5 | 40.5 | 22.3 |

Figure 3I

| # | TAMRA+ macrophages (%) | | | |
| --- | --- | --- | --- | --- |
|  | - | | + | |
|  | Ctrl | Agr-ID | Ctrl | Agr-ID |
| 1 | 45.6 | 55.6 | 67.2 | 88.2 |
| 2 | 40.3 | 55.9 | 65.5 | 78.2 |
| 3 | 45.6 | 60.3 | 63.3 | 85.5 |
| 4 | 43.2 | 61.2 | 57.7 | 86.3 |
| 5 | 47.3 | 52.2 | 66.4 | 80.1 |

Supplementary Fig 1B

| # | Fluorescence intensity | |
| --- | --- | --- |
|  | Ctrl | Agr-ID |
| 1 | 115 | 113 |
| 2 | 143 | 109 |
| 3 | 127 | 112 |
| 4 | 167 | 146 |
| 5 | 138 | 153 |

Supplementary Fig 2A

| # | Number of colonies | |
| --- | --- | --- |
|  | Ctrl | Agr-ID |
| 1 | 130 | 49 |
| 2 | 132 | 133 |
| 3 | 56 | 68 |
| 4 | 966 | 374 |
| 5 | 570 | 88 |

Supplementary Fig 2B

| # | Number of colonies | |
| --- | --- | --- |
|  | Ctrl | Agr-ID |
| 1 | 139 | 59 |
| 2 | 162 | 117 |
| 3 | 87 | 136 |
| 4 | 49 | 88 |
| 5 | 146 | 46 |

Supplementary Fig 2C

| # | Number of colonies | |
| --- | --- | --- |
|  | Ctrl | Agr-ID |
| 1 | 5 | 8 |
| 2 | 7 | 4 |
| 3 | 3 | 4 |
| 4 | 3 | 3 |
| 5 | 1 | 2 |
